# An exploratory randomized controlled trial protocol evaluating Cognitive Behavioral Therapy for Insomnia with adjunct Ayurvedic external therapies (*Siro-pichu* and *Padabhyanga*) in perimenopausal women

**DOI:** 10.3389/fmed.2026.1785264

**Published:** 2026-04-10

**Authors:** Parvathy Unnikrishnan, Devipriya Soman, Sreedevi V, Hemavathi Shivapura Krishnarajabhatt, Sreelekshmi R, Preetha Menon, L. M. Frey, Athira Jacob Thattil

**Affiliations:** 1 Department of Striroga and Prasuti Tantra (Gynaecology and Obstetrics), Amrita School of Ayurveda, Amrita Vishwa Vidyapeetham, Kollam, India; 2 Department of Kayachikitsa, Amrita School of Ayurveda, Amrita Vishwa Vidyapeetham, Kollam, India; 3 Department of Shalyatantra, Amrita School of Ayurveda, Amrita Vishwa Vidyapeetham, Kollam, India; 4 Department of Kayachikitsa, Amrita School of Ayurveda, Kollam, Kerala, India; 5 Amrita Darshanam, Amrita School of Spiritual and Cultural Studies, Kollam, Kerala, India; 6 Department of Cognitive Sciences and Psychology, Amrita School of Social & Behavioral Sciences, Kollam, Kerala, India

**Keywords:** Ayurveda, Cognitive Behavioral Therapy (CBT), insomnia, *Ksirabala Taila*, *Murdhni Taila*, perimenopausal women

## Abstract

**Background:**

Insomnia during perimenopause is a common and clinically significant condition associated with impaired daytime functioning and reduced quality of life. Cognitive Behavioral Therapy for Insomnia (CBT-I) is recommended as first-line non-pharmacological treatment in international guidelines; however, symptom improvement may require several weeks, potentially affecting adherence. External Ayurvedic procedures such as *Siro-pichu* (localized retention of medicated oil over the scalp) and *Padabhyanga* (therapeutic oil massage of the feet) using *Ksirabala Taila* are traditionally described as supportive interventions for sleep regulation. The present study is designed to explore the feasibility and preliminary signal of integrating these procedures with CBT-I.

**Methods:**

This single-center, parallel-group, exploratory randomized controlled trial (CTRI/2025/11/098172) is conducted at the Amrita School of Ayurveda, Kerala, India, with a 1:1 allocation ratio. Sixty-two perimenopausal women aged 45–55 years with subthreshold to moderate insomnia (Insomnia Severity Index [ISI] 8–21) will receive standardized CBT-I for 4 weeks (both groups). The integrative arm will additionally receive *Siro-pichu* for seven consecutive days and *Padabhyanga* for fourteen consecutive days, commencing at baseline. Follow-up continues for 3 months. The exploratory primary outcome is change in ISI score from baseline to Week 4. Exploratory secondary outcomes assess early response (ISI at Week 2), durability of change during follow-up (ISI and PSQI), sleep quality (PSQI), safety (adverse event monitoring), behavioral dependency risk (Severity of Dependence Scale), and treatment adherence (Daily Sleep and Treatment Engagement Diary).

**Discussion:**

This exploratory trial evaluates feasibility, safety, adherence, and preliminary effect-size estimation within a hypothesis-generating framework. It is not designed to establish confirmatory efficacy or superiority.

**Clinical trial registration:**

The trial was prospectively registered with the Clinical Trials Registry–India (CTRI/2025/11/098172).

## 1 Introduction

### 1.1 Background

Insomnia is one of the most common and distressing symptoms reported during the menopausal transition, with a global prevalence of 38–60% among perimenopausal women (1–3). Indian data show similar patterns, with 42–58% of women aged 45–55 years experiencing moderate to severe sleep disturbances (4).

Chronic insomnia during this phase is associated with impaired daytime functioning, mood disturbance, and reduced quality of life. According to the Diagnostic and Statistical Manual of Mental Disorders (DSM-5), chronic insomnia disorder is defined as persistent difficulty initiating or maintaining sleep at least three times per week for more than 3 months, resulting in significant daytime impairment (5–7). In perimenopausal women, fluctuating gonadal hormones, vasomotor symptoms, circadian rhythm alterations, and mood instability contribute to sleep disruption (8–10). However, in this study, insomnia is classified according to chronic insomnia disorder criteria and is distinguished from insomnia secondary to medical or psychiatric illness. This approach allows the evaluation of insomnia within the menopausal transition as a clinical disorder requiring targeted management. Cognitive Behavioral Therapy for Insomnia (CBT-I) is the first-line recommended intervention for chronic insomnia, including in perimenopausal women, owing to its long-term effectiveness in modifying maladaptive sleep-related thoughts and behaviors (11–15). However, CBT-I typically requires 4–6 weeks for noticeable improvement, and early discontinuation rates of 30–40% have been reported (16–19). Safe, culturally acceptable non-pharmacological adjuncts that may support early symptom stabilization warrant exploration. External Ayurvedic procedures such as *Siro-pichu* (localized retention of medicated oil over the scalp) and *Padabhyanga* (therapeutic oil massage of the feet) are traditionally described as supportive interventions for sleep regulation; however, high-quality randomized studies evaluating their integration with CBT-I in perimenopausal insomnia are lacking.

The present exploratory randomized controlled trial is therefore designed to evaluate feasibility, safety, adherence, and preliminary signal detection rather than confirmatory efficacy, with the aim of informing future adequately powered trials.

### 1.2 Literature review

A structured narrative literature review was conducted during protocol development to inform the clinical rationale and selection of outcome measures. Electronic databases including PubMed, Scopus, ScienceDirect, and the AYUSH Research Portal were searched for publications from 2000 to 2025. Search terms included combinations of *“perimenopausal insomnia” “chronic insomnia,” “Cognitive Behavioral Therapy for Insomnia,” “CBT-I,” “Ayurveda,” “Śiro-pichu,” “Padabhyanga,” “Kṣirabala Taila,“integrative sleep therapy.”* Peer-reviewed clinical trials, observational studies, systematic reviews, and relevant narrative reviews published in English were considered. Classical Ayurvedic texts, including (*Caraka Saṃhita, Susruta Saṃhita, Aṣtanga Hrdaya, Astanga Sangraha*) were consulted to identify traditional descriptions of sleep disturbances and *Murdhni Taila (external oil based procedures for head)* and *Abhyanga (therapeutic oil massage)*. Two investigators independently screened titles and abstracts for relevance to perimenopausal insomnia and integrative sleep management. Relevant full texts were reviewed for methodological quality and clinical applicability. This review was conducted as a structured narrative synthesis and was not designed as a systematic review or meta-analysis. The findings informed the feasibility considerations, intervention selection, and outcome framework of the present exploratory trial.

The search covered evidences of interplay between endocrine fluctuations and vasomotor symptomology, evaluating the responsiveness of these physiological stressors to standardized CBT-I protocols in contrast to targeted Ayurvedic external therapies.

The present study aims to explore preliminary between-group differences associated with integrating Ayurvedic external therapies with CBT-I, with findings interpreted within a hypothesis-generating framework rather than to establish confirmatory superiority. CBT-I is recommended as first-line treatment for chronic insomnia by the American Academy of Sleep Medicine (20) and the European Sleep Research Society (21) in terms of correcting maladaptive cognitions, improves stimulus control, and enhances sleep hygiene. Behavioral insomnia management strategies incorporating CBT-I principles are also reflected in Indian clinical practice recommendations. Its use in the present study therefore represents guideline-supported standard care rather than experimental therapy. Its major limitation is delayed onset of benefit, which reduces adherence during the early phase of treatment (11–18).

Many perimenopausal women seek non-pharmacological alternatives to avoid risks associated with hypnotics, such as tolerance, withdrawal, and cognitive impairment. This study evaluates the integration of CBT-I with external, non-systemic Ayurvedic therapies to accelerate clinical improvement and reduce pharmacological reliance.

Classical Ayurvedic literature provides detailed descriptions of sleep disturbances under *Vata* (principle governing movement and nervous system function) predominant conditions.

The menopausal transition is traditionally associated with a physiological shift from *Pitta* (functional principle associated with metabolic regulation) towards *Vata* dominance, predisposing women to symptoms such as *Nidranasa* (disturbance or loss of sleep), *Alpanidra* (reduced sleep duration), and *Nidrabhramsa* (fragmented or disturbed sleep) as described in Caraka Samhita and Astanga Hrdaya (22, 23).

Among Ayurvedic therapies, internal formulations such as *Ashwagandha* (24), *Shatavari* (25), *Mamsyadi Kwatha* (26) and procedures such as *Sirodhara* (27) have been studied in relation to sleep and menopausal symptoms. These interventions are known to exert systemic or pronounced neurophysiological effects and have generally been evaluated as stand-alone therapies. While clinically relevant, their integration with CBT-I poses challenges for trial design because they may act as independent treatments and introduce pharmacological or physiological confounding.

In the present study, external therapies *Siro-pichu* and *Padabhyanga* were selected as adjuncts to CBT-I based on pragmatic, methodological, and ethical considerations. Procedures such as *Sirodhara* require prolonged, resource-intensive administration and are less feasible in outpatient-based care, limiting their suitability for pragmatic trials intended to reflect routine clinical practice. In contrast, *Siro-pichu* and *Padabhyanga* can be delivered in an outpatient.

setting and are easier to integrate alongside counseling-based interventions. *Siro-pichu* and *Padabhyanga* are classical *Vata-*pacifying procedures recommended for calming mind–body instability and promoting sleep (28–32). *Ksirabala Taila*, used in these therapies, is a traditional formulation with Vata-Pitta balancing action and documented neuroprotective and anxiolytic properties. Preclinical studies suggest effects on GABAergic modulation and cortical excitability (33, 34). Limited clinical data are currently documented regarding the role of *Padabhyanga* in sleep disorders, highlighting the need for systematic clinical evaluation in this area (35, 36).

From a methodological perspective, external oleation therapies were chosen as a conservative adjunct, being less likely to function as independent sedative treatments and therefore allow clearer interpretation of whether external Ayurvedic procedures can complement a behavioral intervention. This approach also minimizes systemic pharmacological exposure and reduces concerns regarding herb–drug interactions in a peri-menopausal population. A pilot trial in a limited number of participants suggested improved acceptability and adherence on the combination therapy. On ethical grounds, this supported the integration of these procedures with CBT-I in order to enhance compliance and participant engagement without introducing more intensive or pharmacologically active interventions.

### 1.3 Ayurvedic perspective

Insomnia (ICD-10: F51.0) is mapped in the AYUSH Standard Terminology as AAB-9: *Aswapna* (*Kevala Vata*), indicating its association with *Vata* predominance. Classical descriptions align perimenopausal insomnia with *Vataja Nanatmaja Vikara (disorders attributed to the functional principle governing nervous system)*. Clinical parallels include *Alpanidra* (reduced sleep duration) and *Nidrabhramsa*, (fragmented or disturbed sleep) which correspond closely to sleep disturbances experienced by perimenopausal women. The perimenopausal phase reflects a natural progression toward *Vata* dominance, resulting in disturbed sleep, restlessness, and early awakenings. In this context, *Snigdha* (oleation therapy) and *Samana* interventions (pacifying therapy) are recommended. The intervention is informed by classical Ayurvedic references found in Caraka Samhita and Astanga Hrdaya, where external oil-based head applications (*Murdhni Taila*) and therapeutic oil massage (*Abhyanga*) are described as supportive procedures in sleep disturbances. In this study, these procedures are operationalized as *Siro-pichu* and *Padabhyanga* using *Ksirabala Taila*. *Siro-pichu* and *Padabhyanga* with *Ksirabala Taila* offer traditional *Vata pitta hara* and *Nidrajanana* (sleep inducing) benefits and are considered appropriate supportive therapies for sleep disturbances.

### 1.4 Pharmacological perspective

*Ksirabala Taila* contains documented phytochemicals such as sterols (*β*-sitosterol), alkaloids, and saponins, though percutaneous absorption and isolated clinical effects of these constituents remain uncertain. Literature evidence supports preclinically that it has got a neuroprotective, anxiolytic, and sedative properties (37, 38). Mechanistic explanations in available studies suggest potential modulation of GABAergic pathways, parasympathetic activation, and neurovascular responses contributing to relaxation, reduced cortical excitability, and sleep induction (39, 40). These actions may support early improvement in sleep parameters when used alongside CBT-I.

### 1.5 Need for the trial

Insomnia during the perimenopausal transition significantly affects daily functioning and emotional well-being. While CBT-I is recommended as first-line treatment for chronic insomnia, its structured behavioral format requires sustained engagement over several weeks. The feasibility of integrating culturally contextualized, non-pharmacological adjunct procedures within CBT-I for perimenopausal women has not been systematically evaluated. External Ayurvedic procedures such as *Siro-pichu* and *Padabhyanga* are traditionally used in sleep-related complaints; however, controlled studies examining their role as adjuncts to CBT-I in insomnia for perimenopausal women is lacking. In particular, there are limited data regarding intervention adherence, procedural feasibility in outpatient settings, safety monitoring, and participant acceptability. This trial focuses on women meeting criteria for chronic insomnia disorder during the perimenopausal transition, conceptualizing perimenopause as a physiological context associated with sleep vulnerability rather than as a secondary pathological cause. The present study is designed as an exploratory randomized controlled trial to evaluate feasibility, safety, adherence, and preliminary between-group signal estimation associated with integrating external Ayurvedic procedures with guideline-supported CBT-I. The study is not intended to establish confirmatory efficacy or mechanistic conclusions. Findings are intended to inform methodological refinement and the design of future adequately powered confirmatory trials.

### 1.6 Research hypothesis

This exploratory study hypothesizes that the addition of *Siro-pichu* and *Padabhyanga* with *Ksirabala Taila* to CBT-I may be associated with preliminary differences in insomnia severity and sleep quality compared with CBT-I alone. Findings will be interpreted as hypothesis-generating.

## 2 Study objectives

### 2.1 Exploratory primary objectives

To determine mean reduction in the ISI (41) score after 4 weeks of intervention with CBT-I added with the external administration of *Siro-pichu* and *Padabhyanga* with *Ksirabala Taila* compared to CBT-I alone.

### 2.2 Exploratory secondary objectives

- To determine mean reduction in the Pittsburgh Sleep Quality Index (PSQI) score (42, 43) after 4 weeks of intervention with CBT-I added with external administration of *Siro-pichu* and *Padabhyanga* with *Ksirabala Taila*, compared to CBT-I alone.
- To determine the early onset of therapeutic response within the first 2 weeks of intervention using the ISI score, with a focus on changes in the severity of insomnia.
- To determine changes in insomnia severity during the three-month follow-up period, measured at two-week intervals using the ISI score.
- To determine the sleep quality measured using the PSQI score during the 3 months of follow up.
- To record the occurrence and severity of ADEs associated with the integrative protocol using a validated ADR monitoring checklist.
- To determine the potential psychological or behavioral dependency risk related to the adjunctive Ayurvedic therapies (*Siro-pichu* and *Padabhyanga*) using Severity of dependency Scale during the 3-month relapse prevention follow-up phase.
- To determine treatment adherence in both intervention and control groups using the Daily Sleep and Treatment Engagement Diary.

## 3 Methods

The following section provides detailed operational procedures to enable replication of the trial protocol.

### 3.1 Trial design

The study is designed as a single-center, parallel-group, exploratory randomized controlled trial with a 1:1 allocation ratio intended to generate preliminary estimates of clinical effect and feasibility rather than to establish confirmatory superiority. The primary purpose of this trial is to identify whether the addition of Ayurvedic external therapies to CBT-I produces a clinically observable signal of benefit that warrants further investigation in larger, confirmatory trials. Accordingly, all analyses are framed as exploratory and hypothesis-generating, and findings will be interpreted with caution regarding causal inference. A sham oleation procedure was not implemented because any tactile oil-based scalp or foot application is likely to exert physiological and expectancy effects. Designing a biologically inert yet credible sham was therefore considered impractical and ethically challenging for this exploratory trial. All procedures described; are operationalized within a single outpatient-based clinical setting and implemented by designated study personnel as specified in each subsection to ensure procedural reproducibility.

### 3.2 Reporting standards

This trial protocol is reported in accordance with the SPIRIT 2025 (Standard Protocol Items: Recommendations for Interventional Trials) guideline.

In addition, the manuscript has been aligned with the updated CONSORT 2025 reporting standards where applicable to enhance transparency and consistency with contemporary randomized trial reporting.

### 3.3 Trial settings

The study will be conducted at Amrita Ayurveda Medical College Hospital, a teaching and research hospital located in Clappana, Vallikavu, Kollam, Kerala, India – 690525 which is a constituent unit of Amrita Vishwa Vidyapeetham. Participant recruitment, screening, baseline assessments, and in-person intervention procedures will take place in designated consultation rooms within the Outpatient Department (OPD) of Prasuti Tantra and Striroga and Kayachikitsa. The hospital has established infrastructure for clinical research, including Institutional Ethics Committee oversight, secure document storage facilities, and trained clinical personnel to support protocol implementation.

### 3.4 Study population

Target Population: Perimenopausal women aged 45–55 years, in the general population, who experience chronic sub-threshold to moderate insomnia.

Intended Population: Target population who attend the Outpatient Department (OPD) of Prasuti Tantra and Stree Roga, Kayachikitsa, Shalyatantra, Amrita Ayurveda Hospital, Amrita School of Ayurveda, Kerala, and present with complaints of insomnia or sleep disturbance.

Study Population: The study population comprises women experiencing chronic insomnia disorder during the perimenopausal transition. Insomnia is operationalized as persistent difficulty initiating or maintaining sleep, or early morning awakening occurring at least three nights per week for a minimum of 3 months with associated daytime impairment not attributable to another identifiable medical, psychiatric, neurological, or substance-related condition. Perimenopause is conceptualized as a physiological context associated with sleep disturbance rather than as a pathological secondary cause of insomnia.

To ensure safety and facilitate focused efficacy evaluation, participants must score between subthreshold and moderate insomnia (ISI 8–21).

Eligibility will be determined through structured clinical evaluation conducted by the Principal Investigator or designated Co-investigator during the screening visit. Insomnia diagnosis will be confirmed using DSM-5 criteria, supported by ISI scoring at screening. Perimenopausal status will be verified through menstrual history consistent with STRAW staging criteria (44).

Study duration: Participant recruitment will be conducted over a period of approximately 2 to 3 months within the 6-month study timeline, with potential extension by an additional 1 to 2 months, if required to achieve the desired sample size.

### 3.5 Eligibility criteria

Written informed consent will be obtained from all participants prior to initiation of any study procedures.

#### 3.5.1 Inclusion criteria

Participants must meet all of the following criteria:

- Women aged 45 to 55 years, perimenopausal stage (as per STRAW staging) (44).
- Diagnosis of chronic insomnia disorder consistent with DSM-5 criteria (difficulty initiating or maintaining sleep, or early morning awakening occurring at least three nights per week for a minimum duration of 3 months, with associated daytime impairment).
- Insomnia Severity Index (ISI) score between 8 and 21 at screening.
- Able to understand the study procedures and willingness to comply with CBT instructions.

#### 3.5.2 Exclusion criteria

Participants will be excluded if any of the following are present:

- Known cases of Hypertension, Thyroid disorders, and Diabetes Mellitus.
- Those who require Hormone replacement therapy (HRT) during their menopausal period.
- PHQ-9 Depression scale Score greater than 5 at screening.
- Under medication for any psychological or psychiatric disorders & substance use in the past 30 days.
- Any active medical/ neurological conditions causing sleep disruption.
- No access to mobile phones and internet.
- Engagement in rotating shift work, night shifts, or occupations that involve irregular sleep–wake cycles, or any known lifestyle factors that disrupt normal sleep timing.
- Use of sleep-inducing medications, anti-depressants, anti-psychotics, or sedatives in the past 30 days.
- Body Mass Index (BMI) > 35.

BMI will be measured using a calibrated digital weighing scale and stadiometer at screening. PHQ-9 will be administered in paper format and scored immediately during the screening visit to determine eligibility.

### 3.6 Interventions

#### 3.6.1 Overview

All participants will receive Cognitive Behavioral Therapy for Insomnia (CBT-I) and standardized sleep hygiene counseling through trained therapists using a structured treatment manual. Participants randomized to the Ayurvedic intervention arm will additionally receive the adjunct Ayurvedic regimen during the first 2 weeks of the study, consisting of *Siro-pichu* and *Padabhyanga* using *Ksirabala Taila*. These procedures will have predefined duration, frequency, and monitoring protocols to ensure procedural consistency and reproducibility. Participants in the control arm will receive CBT-I alone for the same study duration. CBT-I sessions will be delivered in a private consultation room within the OPD or via secure telehealth platform as scheduled. Ayurvedic procedures will be demonstrated in person during the baseline visit and subsequently performed at participants’ homes.

#### 3.6.2 Ayurvedic regimen (add-on group)

- *Siro-pichu* (Days 1–7): A sterile cotton pad (approximately 3-inch square and ¼-inch thick) wrapped in gauze is soaked with lukewarm *Ksirabala Taila* and placed on the vertex (anterior fontanelle region). About 10 mL of oil is applied to saturate the pad completely. The pad is secured with sterile bandage cloth wrapped around the head in a standard pattern, and the procedure is performed nightly for 30 min, approximately 1 h before bedtime, for 7 consecutive days. After completion, the area is wiped clean with a dry cloth.
- *Padabhyanga* (Days 1–14): Each session begins with cleansing the feet with lukewarm water. About 10 mL of warmed *Ksirabala Taila* is applied to the plantar aspect of each foot.

Massage includes:

- Thumb pressure to the bases of the toes.
- Gentle stroking and circular movements.
- Stretching of toes.
- Heel massage.
- Circular movements around ankle joints.
- Light calf massage.

Each foot is massaged for at least 5 min, twice daily (morning and night). After the procedure, oil remains on the feet for 30 min before being wiped off with a clean cloth.

##### 3.6.2.1 Training, monitoring and fidelity

On Day 0, a trained Co-Investigator will provide an in-person demonstration at the OPD to standardize oil quantity, application methods, and safety precautions. Participants or caregivers will receive illustrated bilingual pamphlets and video guides. *Ksirabala Taila*, sourced from the GMP-certified manufacturer, must be maintained at a lukewarm temperature prior to application. The designated investigator will verify participant competency during the session using a standardized procedural checklist. Adherence is monitored via Daily Sleep and Treatment Engagement Diary (DSTED) logs and weekly tele-supervision for 10% of the cohort to maintain intervention fidelity.

#### 3.6.3 Cognitive Behavioral Therapy for Insomnia (intervention and control group)

Participants randomized to the control arm will receive Cognitive Behavioral Therapy for Insomnia (CBT-I) alone for 4 weeks. CBT-I will be delivered twice weekly in 45–60-min individual sessions, either in person or via telehealth, by trained and certified counseling psychologists with a minimum of 2 years of clinical experience. All CBT-I therapists will undergo a protocol orientation meeting prior to study initiation to ensure adherence to the standardized CBT-I manual used in this trial. Session content will be documented after each visit using structured therapist notes. Treatment will follow the same standardized CBT-I manual as described, including sleep restriction therapy, stimulus control therapy, cognitive restructuring, sleep hygiene education, and relapse prevention components.

Participants in the control arm will undergo the same follow-up schedule, outcome assessments, adherence monitoring procedures, and safety oversight as those in the intervention arm to ensure methodological parity between groups. A sham oleation procedure was not included, as standardizing a credible placebo tactile intervention was considered impractical in the context of this exploratory trial.

##### 3.6.3.1 CBT-I module structure

A total of eight sessions follow a structured progression:

**Session 1:** Assessment, introduction to CBT-I, review of comorbidities, and instruction on sleep diary use.
**Session 2:** Sleep Restriction Therapy (SRT) and Stimulus Control Therapy (SCT); introduction to the 3P Model of insomnia; setting the initial sleep prescription.
**Session 3:** Sleep hygiene education, addressing barriers to adherence, reviewing sleep schedules.
**Session 4:** Introduction to cognitive therapy principles and strategies.
**Sessions 5–7:** Continued cognitive restructuring, adherence monitoring, adjustment of sleep prescription if sleep efficiency exceeds 90%, review of sleep hygiene.
**Session 8:** Relapse prevention, review of progress, and final recommendations.

##### 3.6.3.2 Educational guidance

All participants will be educated about the benefits of 7–9 h of sleep at night for a midlife adults and relevance of consistent sleep below 6 hours is considered insufficient. This information is for general education and does not affect outcome definitions.

### 3.7 Intervention modifications

All missed sessions and protocol deviations will be documented in the participant’s CRF and reviewed during weekly study meetings.

- Missed Ayurvedic Procedures.

- Missed *Siro-pichu*: Not rescheduled later that night; documented as a protocol deviation; resume next scheduled session.
- Missed *Padabhyanga*: Resume at the next scheduled time; doubling sessions is not allowed.

- Missed CBT-I Session.

- May be rescheduled within the same week or delivered via telehealth within 48 h.

### 3.8 Withdrawal criteria

Participants may be withdrawn from the study under the following circumstances:

- Occurrence of a serious adverse event requiring urgent medical intervention.
- Initiation of pharmacological treatment for insomnia during the study period.
- Emergence of a psychiatric condition requiring immediate clinical management.
- Voluntary withdrawal of consent.

Symptom fluctuation or worsening insomnia severity alone will not constitute automatic withdrawal. Participants experiencing worsening symptoms will undergo clinical evaluation and supportive management. Non-compliance with the intervention schedule will be documented but will not automatically result in withdrawal. All randomized participants will be included in the intention-to-treat analysis wherever feasible.

Withdrawal decisions due to safety concerns will be made by the Principal Investigator in consultation with the Co-Investigator and documented in the CRF.

### 3.9 Protocol adherence criteria

Adherence will be defined using quantitative thresholds to ensure objective assessment.

#### 3.9.1 Adherence to Ayurvedic regimen

- Adherence to *Siro-pichu* and *Padabhyanga* will be defined as completion of at least 80% of the prescribed applications during the intervention phase.
- Completion will be recorded in the Daily Sleep and Treatment Engagement Diary and reviewed during weekly follow-ups.
- Random tele-supervision (10% of participants) will be conducted to support procedural fidelity.

#### 3.9.2 Adherence to CBT-I

- Attendance requirement: participants will be considered adherent if they attend at least six sessions (75–80%) of the scheduled CBT sessions.
- Session documentation: attendance will be recorded by the therapist/facilitator after each session, and non-attendance will be followed up by reminder calls.
- Engagement in sessions: active participation (completion of exercises, discussions, and assignments) will also be documented to assess qualitative adherence.
- Make-up sessions: if participants miss a session, opportunities for catch-up or supplementary guidance will be offered, ensuring minimal loss of therapeutic continuity.

#### 3.9.3 Adherence improving strategies

- Daily DSTED diary entries.
- Weekly telephonic check-ins.
- Visual aids and instructional material for home practice.
- SMS/ WhatsApp reminders during the Ayurvedic regimen and prior to CBT-I sessions.
- Reinforcement during scheduled follow-up visits.

### 3.10 Rescue medications

#### 3.10.1 Mild to moderate symptoms

- *Dhanwantharam Gulika* for mild headache.
- Steam inhalation and *Vyoshadi Vatakam* for nasal congestion.
- *Jeerakarishtam* for light-headedness.
- Coconut oil for mild local skin irritation.
- Warm water and light ambulation for drowsiness.
- Short nap (<30 min), sunlight exposure for daytime sleepiness.
- Breathing exercises and progressive muscle relaxation for irritability and stress.
- Motivational guidance for schedule adjustment difficulties.

#### 3.10.2 Persistent or worsening symptoms

- Referral to a modern medical practitioner.
- Non-pharmacological support measures.

#### 3.10.3 Significant worsening of insomnia

- Intervention paused.
- Referral to sleep specialist or psychiatrist.

All the rescue medications will be sourced from GMP certified manufacturers and the use of all such prescriptions will be documented in the CRF with date, indication, dosage and duration. Although these measures are intended to ensure participant safety and comfort, their use may introduce potential confounding effects on sleep related outcomes and will therefore be considered during interpretation of results.

## 4 Outcome measures

All outcomes in this trial are exploratory in nature and are intended for estimation and hypothesis generation rather than confirmatory hypothesis testing.

### 4.1 Exploratory primary outcome

(Assessed at Week 4—end of intervention phase).

- Insomnia Severity Index (ISI) — change from baseline to Week 4.

- Measurement variable: ISI total score (range 0–28).
- Analysis metric: Mean change from baseline to Week 4; between-group mean difference with 95% confidence interval.

### 4.2 Exploratory secondary outcomes

The following outcomes explore early response, durability of effect, sleep quality, safety, behavioral reliance, and adherence. These analyses are exploratory and descriptive in nature. No adjustment for multiple comparisons is planned.

- Pittsburgh Sleep Quality Index (PSQI) — change from baseline to Week 4.

- Measurement variable: PSQI global score (range 0–21).
- Analysis metric: Mean change from baseline to Week 4; between-group mean difference with 95% confidence interval.

- Early therapeutic response (ISI at Week 2).

- Measurement variable: ISI total score at Week 2.
- Analysis metric: Between-group differences in mean change from baseline with 95% confidence intervals.

- Durability of insomnia severity (ISI during follow-up).

- Measurement variable: ISI total scores at Weeks 6, 8, 10, 12, 14, 16.
- Analysis metric: Mean change from baseline at each time point; descriptive summaries with between-group estimates.

- Sleep quality during follow-up (PSQI).

- Measurement variable: PSQI global scores at Weeks 8, 12, 16.
- Analysis metric: Mean change from baseline; descriptive between-group estimates.

- Occurrence and characterization of clinically relevant events.

- Measurement variable: Adverse events recorded using validated ADR/AE monitoring checklist (45, 46).
- Analysis metric: Frequency and proportion by group; descriptive summaries of severity and relatedness.

- Behavioral dependency risk.

- Measurement variable: Severity of Dependence Scale (SDS) total score (47), assessed through Week 16.
- Analysis metric: Mean change from baseline to Week 16; between-group estimates with 95% confidence intervals.

- Treatment adherence and fidelity.

- Measurement variable: Daily Sleep and Treatment Engagement Diary (DSTED) (48).
- Analysis metric: Percentage adherence and descriptive summaries.

### 4.3 Participant timeline

Enrolled participants follow a sequence of screening, baseline intervention and follow up. During the initial screening (Week 0), the medical officer document demographic details, perimenopausal status (STRAW staging), and chronic insomnia symptoms which is occurring at least three nights per week for the past 3 months. Screening evaluations will include the Insomnia Severity Index (ISI) to determine eligibility, PHQ-9 to rule out depressive symptoms (≤5), review of recent medication use, and assessment of BMI (≤35). Baseline medical history, lifestyle factors (sleep schedule regularity, shift-work exclusion), and technology readiness (phone/internet access) will be verified. *Prakriti* (constitutional profile) assessment and documentation of baseline *Dosa dushti lakshaṇa* (etiopathogenesis with clinical presentation) related to *Nidranasa* (disturbance or loss of sleep) will be completed for descriptive and exploratory analysis. Written informed consent will be obtained at this stage. Following the screening visit, participants will attend a baseline assessment session, during which ISI, PSQI, sleep diary orientation, safety checks, completing the clinical evaluation. Screening and baseline visit may occur on the same day if eligibility is confirmed and consent is obtained. Following which a baseline evaluation is done and, participants are randomized into 1:1 groups. Participants in the add- on arm will receive *Siro-pichu* (7 days) and *Padabhyanga* twice daily (14 days) with demonstration, training and home based adherence monitoring, while both groups receive sleep hygiene education and eight twice-weekly sessions of CBT-I delivered by certified psychologists. Sessions will follow a structured CBT-I manual that includes sleep restriction, stimulus control, cognitive restructuring, relaxation training, and relapse-prevention modules. Adherence will be monitored through attendance logs, sleep diaries, DSTED entries, and telephonic check-ins. Upon completion of the intervention period (at Week 4), participants will attend an assessment post-intervention visit to repeat ISI, PSQI, and other outcome measures.

Durability of treatment effects is evaluated during a post-intervention visit (Week 4) and a 3-month follow-up either in person or via telehealth. Throughout the study, participants will remain in active communication with the research team for support, documentation of adherence, and monitoring of any rescue medication use.

### 4.4 Harms and adverse events monitoring

Harms will be defined as any untoward medical occurrence, whether or not causally related to the intervention, including adverse events (AEs), adverse reactions, serious adverse events (SAEs), and unexpected events. Adverse events will be systematically recorded using the WHO–UMC (World Health Organization–Uppsala Monitoring Center) causality assessment framework.

#### 4.4.1 Monitoring schedule

##### 4.4.1.1 Intervention phase (weeks 0–4)

- Active AE screening will occur at every in-person or telehealth contact.
- A structured ADR/AE checklist will be completed weekly.
- Participants will be specifically asked about new or worsening symptoms.
- DSTED diary entries include prompts for reporting discomfort or unexpected symptoms.

##### 4.4.1.2 Follow-up phase

- AE assessment will be conducted at each scheduled follow-up visit.
- Participants will be instructed to report any adverse events immediately between visits.
- Monthly structured AE review will be performed during follow-up assessments.

#### 4.4.2 Documentation

All AEs will be documented in standardized Case Report Forms including:

- Event description.
- Date of onset and resolution.
- Severity of the event (mild, moderate, severe).
- Assessed relatedness to study intervention.
- Action taken.
- Outcome.

The Principal Investigator will be evaluating each event for severity and causality.

#### 4.4.3 Serious adverse events

Any SAE will be reported to the Institutional Ethics Committee (IEC) within 24 h of awareness Appropriate clinical evaluation, referral and management will be initiated immediately.

In cases where an AE is considered probably or definitely related to the intervention and is of moderate or severe intensity, the intervention will be discontinued for that participant and standard care will be provided. Participants can report issues using emergency contact details provided in the informed consent.

All rescue medications and supportive measures will be documented for outcome interpretation. Due to low risk nature of the trial interventions, no interim harm-related analysis is planned, but continuous safety oversight will be maintained. Participants will be advised to promptly report any discomfort, worsening of insomnia symptoms, headaches, skin irritation, allergic reactions to oils, slips/falls after the intervention or other unexpected physical/ psychological reactions.

### 4.5 Sample size

This study is designed as an exploratory randomized controlled trial intended to estimate preliminary between-group differences and assess feasibility parameters rather than to establish confirmatory superiority.

The sample size was estimated using the standard formula for comparison of two independent means:


$$n=\frac{2s_{p}^{2}{\left(Z_{1-\alpha /2}+Z_{1-\beta }\right)}^{2}}{{\left(\mu_{1}\mu_{2}\right)}^{2}}$$

where $s_{p}$ represents the pooled standard deviation, $\mu_{1}-\mu_{2}$ represents the expected mean difference between groups, $Z_{1-\alpha /2}$ , $Z_{1-\beta }$ correspond to conventional planning parameters.

The expected post-intervention ISI mean in the intervention group (6.9) was informed by pilot observations in perimenopausal women receiving *Siro-pichu* and *Padabhyanga* with *Ksirabala Taila*. The expected post-intervention ISI mean in the control group (10.9) was derived from Ayabe et al. (49), a multicenter randomized controlled trial evaluating CBT-I in adults with pharmacotherapy-resistant insomnia. Although the Ayabe et al. study included both sexes, the majority of participants were women with a mean age of 52 years, supporting its relevance to the present perimenopausal population (49, 50). The anticipated mean difference between groups was therefore approximately 4 points on the ISI scale. A pooled standard deviation of 4.3 was derived from pilot data. Using a two-sided alpha of 0.05 and 90% power as conservative planning parameters with a 1:1 allocation ratio, the required sample size was estimated at approximately 26 participants per group. Allowing for an anticipated attrition rate of 15%, the final planned sample size was set at 31 participants per arm (total *N* = 62). This calculation was applied as a pragmatic planning tool to avoid underestimation of participant numbers and to permit preliminary effect-size estimation. The study is not powered for confirmatory hypothesis testing, and findings will be interpreted within an exploratory, hypothesis-generating.

### 4.6 Recruitment

Recruitment will be facilitated through educational programs, health talks, and distribution of informational posters at community centers and outpatient clinics. This is to attract focus in recognizing difficulty initiating or maintaining sleep, early morning awakening, and non-restorative sleep associated with daytime impairment commonly experienced during the perimenopausal transition. Following these initiatives, preliminary screenings will be conducted using standardized tools, such as the Insomnia Severity Index (ISI), to identify potential participants meeting the study’s symptom profile. Women identified with moderate to severe insomnia symptoms and meeting the preliminary criteria will be invited for a detailed evaluation at the Outpatient Department (OPD) of the Amrita Ayurveda Hospital, Amrita School of Ayurveda, Clappana, Kollam, Kerala, India.

Recruitment into the study will be contingent upon:

1. Confirmation of perimenopausal status through clinical history (as per STRAW staging criteria).
2. Fulfillment of all established inclusion criteria.
3. Exclusion of individuals based on predefined exclusion criteria.
4. Completion of the informed consent process.

Only those participants who meet the criteria and provide written informed consent will be enrolled in the trial. Recruitment will continue until the predetermined sample size is achieved.

### 4.7 Randomization and allocation sequence

#### 4.7.1 Sequence generation

The random allocation sequence will be generated prior to participant enrollment by an independent statistician using R statistical software (version 4.1.5). Block randomization with a fixed block size of 4 will be used to ensure balanced allocation between study arms throughout recruitment. The allocation ratio will be 1:1 (Intervention Group: Control Group). No stratification factors will be applied. The statistician will not be involved in recruitment, intervention delivery, or outcome assessment.

#### 4.7.2 Allocation concealment mechanism

Based on the randomization list, a designated Medical Officer not involved in outcome assessment or statistical analysis will prepare sequentially numbered, opaque, sealed envelopes (SNOSE). Each envelope will contain a card specifying the group allocation. Envelopes will be stored in a locked cabinet within the study office and opened sequentially in strict numerical order. The intervention codes will only be revealed after the completion of all post-intervention assessments or earlier in the event of a medical emergency that requires unblinding.

#### 4.7.3 Implementation and blinding

Participant recruitment and eligibility confirmation will be conducted by the Principal Investigator and designated Co-Investigators at the OPD. After completion of baseline assessments and confirmation of eligibility, the investigator will request allocation from the designated trial coordinator. The next sequential envelope will then be opened to reveal group assignment. Due to the nature of the intervention (CBT-I ± Ayurvedic add-on), participants and Ayurvedic intervention providers cannot be blinded.

The following personnel will remain blinded:

- CBT-I therapists (will deliver sessions without knowledge of group allocation).
- Independent outcome assessor (will administer ISI, PSQI, and follow-up measures).
- Statistician (will analyze anonymized coded datasets).

The Principal Investigator will oversee study conduct and ethical compliance without access to the allocation list. The use of a fixed block size will maintain balance between groups, while the allocation concealment method will prevent selection bias. The independent generation of the randomization sequence will avoid manipulation of group assignments. This structured process ensures allocation concealment until assignment and minimizes selection, performance, and detection bias.

- Role of Principal Investigator (PI): The Principal Investigator will oversee protocol conduct and ethical compliance but will not be involved in allocation concealment, intervention delivery, or outcome assessment.

##### 4.7.3.1 The following personnel will remain blinded throughout the trial

- CBT-I psychologists: Will deliver all CBT-I sessions using a standardized manual without being informed of whether the participant is in the intervention or control group.
- Independent Outcome assessor (Co-investigator): Will conduct ISI, PSQI, sleep diary review, and follow-up assessments while blinded to the allocation to minimize assessment bias.
- Statistician: Will perform data analysis using coded datasets. Unblinding will occur only after completion of the final statistical analysis.

##### 4.7.3.2 Personnel who cannot be blinded (due to study procedures)

- Participants: Cannot be blinded because only the intervention group receives visible Ayurvedic therapies (*Siro-pichu* and *Padabhyanga*), while the control group does not.
- Co-investigators as Ayurvedic intervention providers: Cannot be blinded because they directly administer and train participants in *Siro-pichu* and *Padabhyanga* procedures.
- Medical Officer/Designated Research Staff: Responsible for maintaining the allocation list, distributing intervention kits (*Kṣirabala Taila*), and ensuring allocation concealment. This person will not be involved in outcome assessment or data analysis. This structured approach ensures rigorous randomization, allocation concealment, thereby maintaining methodological integrity and minimizing potential biases throughout the trial.

### 4.8 Data collection and management

#### 4.8.1 Data collection procedures

Study data will be captured via standardized paper-based case record forms (CRFs) and validated outcome instruments. Trained research staff, independent of intervention delivery, will oversee data collection at the Outpatient Department (OPD) during screening and baseline. Subsequent intervention (Weeks 2, 4) and follow-up (Weeks 6–16) assessments will occur in person or via telehealth. Outcome instruments (ISI, PSQI, SDS, DSTED) are self-administered by participants under supervision of trained research staff independent of intervention delivery. Research staff may clarify instructions but will not influence responses (Table 1).

**Table 1:** Data collection methods.

Trial instrument	Purpose	Frequency of measurement	Timepoints of assessment
Insomnia Severity Index	To determine the primary outcome: severity of insomnia symptoms over the preceding two weeks	Every 2 weeks	Baseline (Week 0), Week 2, Week 4 (end of intervention), Week 6, Week 8, Week 12, Week 14, Week 16 (end of follow-up)
Pittsburgh Sleep Quality Index	To evaluate the overall sleep quality and its domains, including latency, duration, efficiency over the past month	Monthly	Baseline (Week 0), Post- treatment (Week 4), Follow-up Months 1, 2, and 3
ADR Monitoring Checklist	To identify and document any adverse events or reactions related to the integrative intervention	Ongoing monitoring at each contact point	Weekly during intervention (Weeks 0– 4), and monthly during follow-up (Months 1–3)
Severity of dependency Scale	To detect any signs of behavioral or psychological dependency on *Śiro-pichu* and *Pādābhyanga* therapies	Twice during the study	Post-intervention (Week 4), End of follow-up (week 16)
Daily Sleep and Treatment Engagement Diary	To assess adherence and consistency in following the prescribed intervention protocol	At the end of intervention and follow up	Week 4 of intervention, Week 16 of follow up

Official Malayalam versions of ISI and PSQI were obtained from ePROVIDE–MAPI Research Trust (COA request submitted 08-Aug-2025; approval date: 01 September 2025). Instruments will be administered in accordance with licensing terms. Table 1 shows the data collection methods in detail.

Sleep diaries will record total sleep time (TST). For descriptive summaries, TST will be categorized as optimal (7–9 h), short (<6 h), or long (>9 h). Classification is for characterization only and will not determine treatment response.

#### 4.8.2 Quality control and verification

Completed CRFs will be reviewed on the same day by supervising research staff. Within 24 h, the Principal Investigator or designated Co-Investigator will verify completeness, internal consistency, and protocol adherence.

ADR/AE checklists and DSTED entries will be reviewed at each contact. Data verification will occur before entry into the electronic database.

#### 4.8.3 Documentation and confidentiality

All study documentation will be maintained in paper format. Prior to enrolment, potential participants will receive the PIS and ICF in English and Malayalam. Written informed consent will be obtained before initiation of any study procedures. Each participant will be assigned a unique study ID code used on all CRFs and instruments. Personal identifiers will be recorded only on consent forms and screening logs and stored separately from analytical datasets. Paper documents will be stored in locked cabinets within the Department of Prasuti Tantra and Striroga. Access will be restricted to authorized study investigators.

#### 4.8.4 Retention

Participation in this study is entirely voluntary. Participants may withdraw at any time without penalty or loss of entitled care and this will be documented in study records. Participants who withdraw will not undergo any additional data collection or follow-up assessments. Data collected prior to withdrawal will be retained for analysis unless the participant explicitly requests removal, subject to regulatory and ethical guidelines. Efforts will be undertaken, where appropriate and ethically permissible, to minimize unnecessary dropouts while respecting participant autonomy. All study records will be retained for a minimum of 5 years following study completion in accordance with institutional policy and Indian Council of Medical Research (ICMR) guidelines.

#### 4.8.5 Data management

Data will be collected using standardized paper-based case record forms and validated outcome assessment instruments completed by participants during scheduled visits. The collected data will subsequently be entered into a password-protected Microsoft Excel spreadsheet maintained on a secure institutional computer. The Excel file will be stored within an encrypted folder with restricted user access, limited to the Principal Investigator and the designated Co-Investigator. A separate master linkage file containing participant identifiers will be maintained independently from the analytical dataset in a secured location accessible only to these investigators. The Principal Investigator will oversee all data management procedures and periodically verify the completeness and accuracy of entries. The Co-Investigator will be responsible for entering outcome scores, follow-up notes, and documented observations into the Excel sheet while maintaining blinding where applicable. All participant data will be coded using unique study identification numbers. Only validated instrument scores and documented clinical observations will be included in the final analytical dataset. The Statistician, who remains blinded to group allocation, will receive an anonymized dataset exported from the Excel file for analysis according to the pre-specified statistical analysis plan. Regular backups will be maintained on secure institutional servers to ensure data integrity and prevent data loss. Role-based access restrictions will be implemented throughout the study to maintain confidentiality.

### 4.9 Exploratory statistical analysis plan

Baseline demographic and clinical characteristics; including age, duration of insomnia, comorbidities, and medication history will be summarized separately for the intervention and control groups. Continuous variables will be reported as mean ± standard deviation (SD) for normally distributed data or as median with interquartile range (IQR) for non-normally distributed data. Categorical variables will be presented as frequencies and percentages. Baseline group comparisons will be explored using Independent *t*-tests or Mann–Whitney U tests for continuous variables and Chi-square or Fisher’s exact tests for categorical variables, as appropriate. These comparisons are descriptive and intended to characterize the sample rather than to establish statistical equivalence. The exploratory primary and secondary outcomes are defined in detail in the Outcome Measures section. Analyses are estimation-focused and hypothesis-generating in nature.

#### 4.9.1 Exploratory primary outcome analysis (week 4)

Change from baseline to Week 4 in ISI score will be summarized for each group. Between-group differences in mean change will be estimated using Independent *t*-tests for normally distributed data or Mann–Whitney U tests for non-normally distributed data. Effect sizes with 95% confidence intervals will be reported. *p*-values will be presented for descriptive purposes and interpreted cautiously within the exploratory framework.

#### 4.9.2 Exploratory secondary outcome analysis

Follow-up ISI and PSQI scores beyond Week 4 will be summarized descriptively. Data will be presented as mean ± SD or median (IQR), and graphical trends over time will illustrate within-group patterns. No formal repeated measures ANOVA or confirmatory longitudinal modeling is planned. These analyses are exploratory and not adjusted for multiple comparisons. Sleep-duration categories (<6 h, 7–9 h, >9 h) will be used for descriptive summaries and exploratory subgroup analysis. No confirmatory inference will be based solely on duration categories. Adverse events will be summarized by frequency and proportion in each group. Between-group comparisons will be explored using Chi-square or Fisher’s exact tests. Severity of Dependence Scale (SDS) scores and adherence percentages will be summarized descriptively and compared between groups using appropriate parametric or non-parametric tests. Findings will be interpreted as exploratory.

### 4.10 Handling missing data & population analysis

- Primary analysis: Intention-to-treat (ITT).
- Missing data management: Multiple imputation will be used if missing exceeds 10% and a per-protocol analysis will be done alongside ITT analysis to assess robustness.

### 4.11 Subgroup analyses

Exploratory subgroup analyses will be conducted to examine whether treatment effects vary across key demographic and clinical characteristics. Subgroups will include age categories (45–50 vs. 51–55 years), BMI classifications (normal vs. overweight), and baseline insomnia severity (sub-threshold vs. moderate insomnia based on ISI scores). In addition, Ayurvedic baseline assessments—including *Prakriti* (individual body constitution) profiling and documentation of *Dosha dushti lakshana (symptoms associated)* associated with *Nidranasa*—will be incorporated to explore potential correlations between *Dosha* predominance and insomnia severity. These analyses are exploratory in nature and intended to generate hypotheses for future confirmatory studies. All statistical analyses will be performed using R version (4.1.5).

### 4.12 Interim analysis

Interim analysis is not planned for this study. The intervention is a non- pharmacological behavioral therapy (Cognitive Behavioral Therapy for Insomnia) posing minimal risk to participants. The study duration and sample size are such that interim analysis is not required. All analyses will be conducted after the completion of data collection.

### 4.13 Auditing

Being a minimally risk posing intervention, a formal independent audit is not planned. Trial is conducted entirely within a clinical setting with routine monitoring of trial conduct and data quality by the Principal Investigator (PI) and the study team through regular team meetings and review of source documents, case report forms, and consent forms. The Institutional Ethics Committee (IEC) may perform audits or monitoring visits at its discretion to ensure compliance with the approved protocol, ethical standards, and applicable regulations. Although any audit requests from the IEC will be fully facilitated by the study team.

### 4.14 Safety

All adverse events (AEs) and serious adverse events (SAEs) will be systematically documented in the Case Report Form (CRF) using a predefined Adverse Drug Reaction (ADR) page. Each entry will capture the event description, diagnosis (if available), onset and resolution dates, severity (mild, moderate, or severe), action taken with the study drug (none, temporarily interrupted, or permanently withdrawn), outcome (resolved, resolved with sequelae, or not resolved), and the assessed relationship to the study drug (definite, probable, possible, unlikely, not related, or not assessable). The Principal Investigator and Co-Investigator will independently evaluate causality, taking into account the temporal relationship with the study drug, the participant’s clinical course, medical history, and concomitant medications. All adverse events will be actively managed by the study investigators with access to on-site medical services at the study institution. Escalation will follow institutional clinical pathways, including specialist referral when indicated. Costs of medical management for trial-related adverse events will be borne by the institution in accordance with ethical guidelines.

Events occurring after a participant exits the study will not be routinely reported unless the investigators consider a reasonable likelihood of relation to the study drug, psychotherapy, or study procedures. This systematic documentation ensures continuous monitoring and prioritization of participant safety throughout the trial.

## 5 Discussion

Insomnia during the perimenopausal transition represents a clinically significant concern, with multifactorial contributors including hormonal fluctuations, vasomotor symptoms, and age-related physiological changes. Cognitive Behavioral Therapy for Insomnia (CBT-I) is internationally recognized as the first-line non-pharmacological treatment for chronic insomnia, including in midlife women. However, early disengagement and the gradual onset of symptomatic improvement are recognized practical challenges in routine implementation. These considerations provide a rationale for exploring adjunctive strategies within a structured therapeutic framework.

External Ayurvedic procedures such as *Siro-pichu* and *Padabhyanga* using *Ksirabala Taila* are traditionally described for sleep-related disturbances including *Nidranasa* and *Alpanidra*. Preclinical and traditional literature describe potential calming and *Vata*-modulating properties of these procedures. In the present protocol, these theoretical considerations serve only as background rationale. The study is not designed to test biological mechanisms, and any reference to possible pathways such as parasympathetic modulation or neurotransmitter effects remains hypothesis-generating and indirect.

Recent comparative studies examining combinations of CBT-I with pharmacological agents (51–53) report improvements in insomnia severity indices and subjective distress. While the populations and interventions in those studies differ from the present perimenopausal cohort, these findings reflect broader research interest in multimodal insomnia management (54, 55). The present exploratory trial contributes to this field by evaluating a structured behavioral therapy in combination with traditional external procedures within a defined outpatient setting.

Importantly, this study does not aim to establish efficacy, superiority, or causal mechanisms. Rather, it evaluates feasibility, safety, adherence, and preliminary signal estimation associated with integrating external Ayurvedic therapies with CBT-I. The focus remains on operational practicality, participant acceptability, and methodological refinement.

Methodologically, the trial incorporates standardized diagnostic criteria (DSM-5), validated outcome measures (ISI, PSQI), structured CBT-I delivery by trained psychologists, predefined adherence thresholds, and systematic adverse event monitoring. These design elements aim to ensure internal consistency and reproducibility within an exploratory framework.

Findings from this study will be interpreted cautiously and are intended to inform the design of future adequately powered confirmatory trials.

## 6 Limitations

This exploratory trial has several methodological and practical limitations. The absence of a sham oleation arm limits differentiation between tactile, sensory, and specific intervention-related effects. Ethical and practical considerations; particularly the challenge of standardizing a credible inert sham made such an arm unfeasible within this exploratory design. Although a formal two-group sample size calculation was used for planning purposes, the study is not powered for confirmatory hypothesis testing. The sample size is intended to provide preliminary effect-size estimation with reasonable precision rather than to establish superiority. Accordingly, findings must be interpreted as hypothesis-generating. The use of rescue medications may introduce potential confounding effects on sleep-related outcomes and will therefore be considered during interpretation. Objective sleep measures such as actigraphy were not included due to logistical and funding constraints; therefore, validated psychometric instruments (ISI, PSQI) are used for outcome assessment. The Severity of Dependence Scale is applied as an exploratory behavioral proxy and is not validated for non-pharmacological procedures; its findings will be interpreted cautiously. Restriction to women with subthreshold and moderate insomnia enhances safety and reduces heterogeneity but may limit generalizability. Exclusion of common medical comorbidities further reduces external validity. Additionally, this is a single-center study with modest sample size and limited follow-up duration; thus, results should be regarded as preliminary.

## 7 Conclusion

This exploratory randomized controlled trial evaluates the feasibility, safety, adherence, and preliminary signal estimation associated with integrating *Siro-pichu* and *Padabhyanga* with CBT-I for perimenopausal insomnia. The study is not designed to determine causality or establish efficacy. Findings will be interpreted within a hypothesis-generating framework and are intended to guide the design of future larger, multi-center, adequately powered confirmatory trials.

## 8 Protocol and statistical analysis plan

- The full trial protocol and statistical analysis plan are available from the corresponding author upon reasonable request.
- The protocol version submitted for ethical approval constitutes the current reference document for trial conduct.
- Any protocol amendments will be documented, dated, and updated in the trial registry as required.

### 8.1 Protocol amendments

Any protocol modifications necessitated by safety or operational concerns will be submitted to the IEC for approval before implementation.

### 8.2 Risk–benefit assessment

The combined intervention (CBT-I with *Siro-pichu* and *Padabhyanga*) is considered low risk. Possible minor risks include transient discomfort, drowsiness, mild skin irritation from medicated oils, or emotional distress during CBT-I. Safety instructions will be provided to minimize risks such as slipping after oil application. Adverse events will be recorded using the WHO-UMC ADR framework and managed promptly. Serious adverse events will be reported to the IEC within 24 h. Potential benefits include improved sleep onset, duration, and quality, reduction in insomnia severity, and better overall wellbeing.

### 8.3 Data handling and monitoring

The Principal Investigator will ensure adherence to the protocol and ethical compliance. An independent monitoring team appointed by the IEC will conduct periodic oversight. Any protocol deviations will be documented and reported.

### 8.4 Ancillary and post-trial care

All participants will receive CBT-I as part of standard non-pharmacological care for insomnia. If the combined intervention demonstrates significant benefit, participants in the control group will be offered the Ayurvedic regimen post-trial. Travel expenses for study visits will be reimbursed, and refreshments will be provided without constituting undue inducement. In the event of research-related injury, compensation will be provided according to ICMR guidelines and institutional policy.

### 8.5 Confidentiality

All study-related data will be securely maintained at the study site. The participant information will be securely maintained in locked cabinets in restricted access zones. All reports, data collecting, and administrative documents shall be designated by a coded ID number solely to preserve participant confidentiality. All documents, including names or other personal identifiers, including locator forms and informed consent forms, will be retained separately from study data designated by code number. All local databases will be safeguarded with password-protected access systems.

### 8.6 Access to data

Access to identifiable study data will be restricted to the Principal Investigator and Co-Investigator for the purposes of study oversight and data verification. All other study personnel, including therapists and outcome assessors, will have access only to participant-specific information necessary for their assigned role. The independent assessor and statistician will receive anonymized datasets and will remain blinded to group allocation during analysis. No external parties, including product manufacturers or third-party organizations, will have access to identifiable or analytical datasets. Data will not be shared outside the research team without prior ethical approval and, where required, participant consent. The Principal Investigator retains final responsibility for data integrity and access control throughout the study.

### 8.7 Dissemination policy

#### 8.7.1 Trial results

The investigators are committed to the transparent and responsible dissemination of the trial results. Upon completion of the study and analysis of data, the findings will be communicated through multiple channels to ensure that participants, healthcare professionals, the scientific community, and the general public are adequately informed. Once the study is completed, it will be submitted to peer-reviewed national or international scientific journals, regardless of whether the outcomes are positive, negative, or inconclusive. Additionally, results will be presented at relevant academic conferences and seminars to facilitate knowledge exchange among clinical and research professionals.

Participants who express interest will be informed of the overall study findings in lay language through written summaries or verbal debriefings. The research outcomes may also be registered and shared through publicly accessible clinical trial registries or results databases, following ethical and regulatory requirements. There are no publication restrictions imposed by the Amrita School of Ayurveda. However, all publications and public disclosures will undergo review by the principal investigator and ethics committee to ensure accuracy, integrity, and compliance with institutional and ethical standards. Any data shared publicly will be de-identified to protect participant confidentiality.

## Data Availability

De-identified individual participant data will be made available after publication of the primary study results. Shared materials may include the anonymized dataset, data dictionary, and statistical analysis code. Data access will be provided upon reasonable request, subject to institutional policies, ethical approval conditions, and data protection regulations. Data will be shared only for academic and research purposes.

## Author’s note

Devipriya Soman is the corresponding author for public queries. Parvathy Unnikrishnan is the corresponding author for scientific queries.
